# β-Carotene: Preventive Role for Type 2 Diabetes Mellitus and Obesity: A Review

**DOI:** 10.3390/molecules25245803

**Published:** 2020-12-09

**Authors:** Gabriela Marcelino, David Johane Machate, Karine de Cássia Freitas, Priscila Aiko Hiane, Iriani Rodrigues Maldonade, Arnildo Pott, Marcel Arakaki Asato, Camila Jordão Candido, Rita de Cássia Avellaneda Guimarães

**Affiliations:** 1 Post Graduate Program in Health and Development in the Central-West Region of Brazil, Federal University of Mato Grosso do Sul, Campo Grande 79070-900, Brazil; gabi19ac@gmail.com (G.M.); priscila.hiane@ufms.br (P.A.H.); cahjordao@gmail.com (C.J.C.); rita.guimaraes@ufms.br (R.d.C.A.G.); 2 Group of Spectroscopy and Bioinformatics Applied Biodiversity and Health (GEBABS), Graduate Program in Science of Materials, Federal University of Mato Grosso do Sul, Campo Grande 79070-900, Brazil; machatedavidjohanemachate@yahoo.com.br; 3 Laboratory of Food Science and Technology, Brazilian Agricultural Research Corporation (Embrapa Vegetables), Brasília 70275-970, Brazil; iriani.maldonade@embrapa.br; 4 Laboratory of Botany, Institute of Biosciences, Federal University of Mato Grosso do Sul, Campo Grande 79070-900, Brazil; arnildo.pott@gmail.com; 5 Faculty of Medicine, Federal University of Mato Grosso do Sul, Campo Grande 79070-900, Brazil; marcel_arakakiasato@hotmail.com

**Keywords:** oxidative stress, dyslipidemia, antioxidants, insulin resistance

## Abstract

Carotenoids are vital antioxidants for plants and animals. They protect cells from oxidative events and act against the inflammatory process and carcinogenesis. Among the most abundant carotenoids in human and foods is β-carotene. This carotenoid has the highest level of provitamin A activity, as it splits into two molecules of retinol through the actions of the cytosolic enzymes: β-carotene-15,15′-monooxygenase (β-carotene-15,15′-oxygenase 1) and β-carotene-9′,10′-dioxygenase (β-carotene-9′,10′-oxygenase 2). The literature supports the idea that β-carotene acts against type 2 diabetes mellitus, cardiovascular diseases, obesity, and metabolic syndrome. Due to the many processes involved in β-carotene biosynthesis and metabolic function, little is known about such components, since many mechanisms have not yet been fully elucidated. Therefore, our study concisely described the relationships between the consumption of carotenoids, with emphasis on β-carotene, and obesity and type 2 diabetes mellitus and its associated parameters in order to understand the preventive role of carotenoids better and encourage their consumption.

## 1. Introduction

Carotenoids are an extensive group of natural pigments present in fruits and vegetables that are responsible for light absorption and have an essential role in the photosynthetic process [1,2]. They are hydrocarbons and can be represented by two main groups: carotenes (nonpolar) and xanthophylls (polar oxygenated) [3]. Vitamin A is a term for carotenoids that have trans-retinol biological activity. In organisms, two of the main precursors to this vitamin are retinyl esters (preformed vitamin A) and carotenoids, which are the β-ring (provitamin A) [1,4,5].

Vitamin A deficiency is one of the most prevalent diseases caused by avitaminosis and is a worldwide public health problem, affecting mainly developing countries. The disease is caused by the low ingestion of food sources of vitamin A, inadequate intestinal absorption, or increased excretion [6,7]. Among the strategies adopted to improve the status of carotenoids are a balanced diet, incentive to breastfeed newborns, supplementation, and food fortification [6]. Among the carotenoids most utilized in supplementation are lutein, zeaxanthin, and β-carotene [8].

Beta-carotene (or β-carotene), belonging to the group of carotenes, is one of the most abundant carotenoids in foods and is also found in the human organism. Approximately 17% to 45% of ingested β-carotene is intact in organisms, suggesting its high bioavailability—that is, its high absorption and utilization capacity [9]. Studies have pointed out that β-carotene acts as a protector against various diseases, such as type 2 diabetes mellitus (T2DM), cardiovascular disease, obesity, and metabolic syndrome (MetS) [10,11,12].

**Figure 1:**
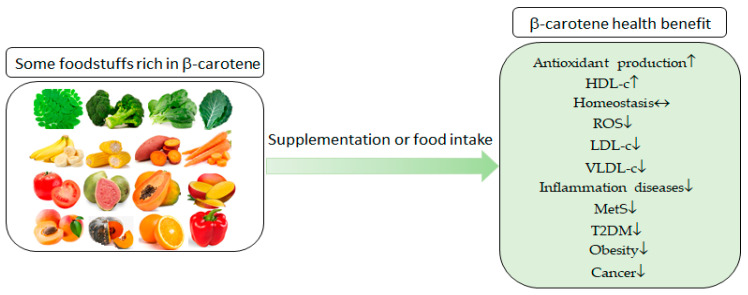
The roles of β-carotene consumption in the prevention of several metabolic diseases and health promotion. HDL-c—high-density lipoprotein cholesterol, ROS—reactive oxygen species, LDL-c—low-density lipoprotein cholesterol, VLDL-c—very-low-density lipoprotein cholesterol, MetS—metabolic syndrome, and T2DM—type 2 diabetes mellitus.

Among the results associated with the consumption of β-carotene are a reduction in the size of adipocytes and body adipose tissue; reductions in proinflammatory markers, low-density lipoprotein cholesterol (LDL-c), and very-low-density lipoprotein cholesterol (VLDL-c); and an increase in high-density lipoprotein cholesterol (HDL-c) [13,14,15,16]. In addition, they can improve insulin resistance and preserve insulin receptors [14,16]. These actions occur to control oxidative stress, which is involved in all of the mentioned diseases [17]. The benefit of consuming a diet rich in β-carotene is correlated with prevention and health promotion, as summarized in Figure 1.

Therefore, we aimed to concisely describe the relationships among the consumption of carotenoids, with emphasis on β-carotene, and obesity and T2DM and its associated parameters, in order to understand the preventive role of carotenoids and encourage their consumption.

## 2. β-Carotene: General Characteristics

Carotenoids are an extensive group of natural pigments that confer different colors varying from yellow to red to plants and foods [1]. In foods, the color intensity is proportional to the content of carotenoids. The Carotenoids Data Base Information [18] cites more than 1181 catalogued natural carotenoids distributed in 700 different organisms; other reports cite only 600 natural compounds, indicating the wide existing variability [1,19].

In plants, carotenoids are responsible for light absorption due to the presence of conjugated double bonds in their structures [2]. The conjugated polyene chromophore is responsible for the capture and absorption of light, acting in coloration, and it exerts photoprotector activity [20]. In general, carotenoids are a class of hydrocarbons derived from eight isoprenoid units forming a 40-carbon polyene chain that can contain terminal groups or be complemented with oxygenated functional groups [1]. Although plants only produce C-40, some organisms are capable of synthesizing carotenoids shorter (30 carbons) or longer chains (45 or 50 carbons) in lower contents [11].

β-Carotene is the most abundant carotenoid in foods. It is found in carrots, pumpkins, and sweet potatoes, among others. Its concentration in foods is variable and is influenced by different factors. Among them are the species, degree of ripening, environmental and growing conditions, sun incidence, storage, and the type of processing the food is subjected to [21]. In addition, β-carotene has been demonstrated to be more bioavailable when dispersed in fats compared with plant structures in natura [22].

Structurally, carotenoids are defined as polyenes with 11 conjugated double bonds and two β-ionone rings on each end [9]. The conjugated double-bond conformation makes them more susceptible to isomerization and oxidation, causing degradation. In addition, the conformation of β-carotene directly affects its conversion into vitamin A and its actions in the organism [23,24].

This carotenoid presents the highest provitamin A activity, since it splits into two retinol molecules through the cytosolic enzymes β-carotene-15,15′-monooxygenase or β-carotene-15,15′-oxygenase 1 (BCMO1) and β-carotene-9′,10′-dioxygenase or β-carotene-9′,10′-oxygenase 2 (BCMO2) [12,13,25]. BCMO1 is a cytosolic enzyme that is involved in the production of retinol acting on the 15′,15′ bond, where it oxygenates two molecules of a retinal, a process that is considered to represent symmetric cleavage. This enzyme acts only in carotenoids with provitamin A activity, such as β-carotene, which is cleaved to all-trans-retinal compounds and, later, gives rise to retinoic acid and all-trans-retinol compounds [26].

However, BCMO2, considered an eccentric cleaved compound of the β-carotene molecule at positions 9 and 10, initially forms β-10′-apocarotenal and then 10,10′-apocarotene-dialdehyde, which, when oxidized, become an all-trans-retinoic acid, an active form of vitamin A. However, little is known about this process. This enzyme is mainly found in the mitochondrial membrane, being less specific than BCMO1 [13,25,27]. The bioavailability of β-carotene is variable. It can reach up to 90% of what is consumed, and its potential for absorption increases in fatty means when compared with plants in natura, as mentioned previously [22,28]. Bioavailability is defined as the fraction available for utilization in various physiological functions after food consumption [29].

In the case of β-carotene, its bioavailability can be affected by the amount consumed, the organism’s needs, storage place, the genetic conditions of the plant, and even by interactions with other nutrients [1,30]. Cooking food at adequate temperatures or grinding is also reflected in an increase in the bioavailability of β-carotene, since it becomes more accessible for absorption in the intestinal lumen [3,12,21,31]. 

In a study evaluating carrots under high-pressure homogenization, it was observed that the bioavailability of β-carotene increased. This was attributed to structural changes that allowed a greater exposure of particles and, thus, better absorption [31]. The temperature range is also an important point for analysis. Temperatures close to 125 °C have been demonstrated to make β-carotene more bioavailable. However, temperatures in the range of 95–115 °C do not present the same result, suggesting that β-carotene is still bound in the cell structures [30].

In enterocytes, the nonmetabolized part is incorporated into chylomicrons, VLDL-c, intermediate-density lipoprotein cholesterol (IDL-c), and LDL-c, which are responsible for their transport, facilitating the entrance into tissues [5,9]. Of these, LDL-c is considered to be the principal transporter responsible for 60–70% of the transport of serum β-carotene to the tissues. In this lipoprotein, β-carotene is stored inside, which is in contrast to the group of xanthophylls that are transported via HDL-c [3,27].

Regarding its activity, β-carotene is considered to be a potent chelator of singlet oxygen and reacts with different species of free radicals (e.g., hydrogen peroxide). The conjugated double chain is responsible for eliminating singlet oxygen, and the higher the number of conjugated bonds, the greater the capacity to eliminate oxygen [1,2]. It quickly picks up singlet oxygen, especially when associated with lycopene, and it has been suggested that β-carotene is capable of eliminating up to 1000 singlet oxygen molecules and then returning to its initial state at the end [12,22].

The ways in which β-carotene and other carotenoids act on these reactive oxygen species or on singlet oxygen itself can involve physical or chemical processes. Physical processes include the transfer of energy between reactive molecules to β-carotene—this process being the most commonly observed—whilst chemical processes include chemical reactions between carotenoids and oxidative species that are formed after cleavage, as is the case with apocarotenals [32].

Carotenoids also stabilize cell membranes against lipid oxidation and decrease the release of lactate dehydrogenase. Furthermore, they alter the pathways related to the inflammatory process and oxidative stress [1,17,33]. In addition, it has been mentioned that β-carotene can act synergistically with other antioxidant compounds, such as tocopherol. When combined, the two can inhibit lipid peroxidation [2].

Its action against free radicals also occurs under low pressure [12]. On the other hand, β-carotene, like other carotenoids, is sensitive to degradation and oxidation, and under certain conditions, it can act as a pro-oxidant [25]. Under high oxygen pressure, this carotenoid produces an unstable intermediate component that reacts with unsaturated fatty acids and initiates the oxidative process, producing reactive oxygen species (ROS) such as epoxides and carbonyls [34]. In addition, when in excess, it can also act as a pro-oxidant [21,22].

It has also been shown that β-carotene can alter the pathways involved in the inflammatory process and oxidative stress by inhibiting cell transcription factors, such as nuclear factor-kappa β (NF-κβ) and inflammatory cytokines, that are also involved in the distribution of adiposity and insulin resistance [10,14,33].

In general, the literature reports that β-carotene is protective against the development of diseases linked to oxidative stress, as it can act against insulin resistance, dyslipidemia, weight loss, degenerative diseases, and some types of cancer, among others [10,11,35].

## 3. Obesity

Obesity is a disease characterized by an increase in fat deposits that can be caused by the atrophy of adipocytes, as well as by its quantitative increase. Among its risk factors, we can mention the dietary factor, in which the individual has excessive energy consumption for their energy expenditure [10,36].

Among other factors related to its development, we can mention existing flaws in the fat oxidation process, genetic factors, and environmental factors [37]. In addition, obesity is related to the development of other comorbidities associated with inflammation, insulin resistance, and dyslipidemia [10,36].

Adipocyte hypertrophy, characteristic of the disease, causes a decrease in the production of adiponectin and, thus, stimulates the development of the inflammatory process and reduces insulin sensitivity [15,38]. Adiponectin is a bioactive protein secreted by adipocytes, which is found mainly in subcutaneous adipose tissue. It has a direct role in regulating insulin resistance, in addition to acting on cardiovascular diseases and MetS. β-carotene is described as an agent that is capable of increasing its expression and secretion, thereby improving its insulin sensitivity [38].

Adipose tissue is considered an endocrine organ, because it secretes adipokines, such as monocyte chemoattractant protein-1 (MCP-1), which are responsible for recruiting monocytes into tissues [39,40]. However, when in excess, fat is responsible for the death of cells in local tissues due to lower blood flow and the infiltration of macrophages, causing inflammatory conditions. Macrophages stimulate the production of cytokines with proinflammatory activity, such as interleukin (IL)-1β, IL-6, and tumor necrosis factor-alpha (TNF-α), which also play roles in the development of insulin resistance [10,41,42,43,44]. These cytokines initiate the inflammatory process in adipose tissue, which later spreads to other parts of the body [42].

It is also possible to find the enzymes BCMO1 and BCMO2 in this tissue, which are related to the metabolism of carotenoids, as described previously [10,44]. These enzymes have been demonstrated to have an essential role in the control of adipose tissue increase and dyslipidemia, as well as regulating the concentrations of carotenoids in adipose tissue, especially subcutaneous tissue [4,9,26,36].

The carotenoids are stored in the adipose tissue. β-carotene and lycopene are the main available fractions, representing one-third and half of all carotenoids, respectively [10]. Adipose tissue holds around 80–85% of the β-carotene available in the organism; the rest stays in the liver (8–12%) and muscles (2% to 3%) [28]. Such data were obtained in a study showing that the liver and adipose tissue are the main storage sites of β-carotene [45]. β-Carotene is stored inside the adipocytes together with triglycerides and can be demanded when necessary to maintain homeostasis in the body [43,46].

β-Carotene also acts within mature adipocytes, helping to control lipid metabolism and regulate oxidative stress and the production of inflammatory mediators. In addition, this carotenoid can inhibit and, therefore, slow down the conversion of preadipocytes to mature adipocytes [47,48]. It is worth pointing out that β-carotene also can suppress cell differentiation in the adipocytes and act in the different phases of its life cycle [10,41].

In a study on female C57/BL6SVJ, it was observed that the supplementation of β-carotene (150 mg/day) for 14 weeks reduced the area of adipocytes and, as a consequence, reduced the body adiposity [13]. The literature reports that β-carotene is a precursor to apocarotenoids, which have the capacity to modulate the physiology of adipocytes through the production of β-apo-14′-carotenal [10,13,46].

β-Carotene also has the capacity to regulate lipogenesis through suppression of the peroxisome-proliferator-activated receptor (PPAR)-y, stimulating a process contrary to what occurs in obesogenic diets and the genetics involved in obesity [38]. PPAR-y belongs to the nuclear hormone receptor superfamily. It is responsible for maintaining the phenotype of adipocytes, controlling their differentiation and stimulating their hypertrophy under a high-fat diet. Thus, when it is present in low quantities in the organism, the mobilization of fats occurs [36,48].

Thus, it is possible to observe that carotenoids can influence the concentration of body fat, since it has been reported that the higher the consumption of carotenoids or their greater their concentration in the body, the lower the body fat content [10]. Carotenoids act directly on adipose tissue, controlling adiposity and fat stores in the diet [36]. A cross-sectional single-center study evaluating 374 adult men found a direct correlation between the intake of carotenoids and weight reduction. With an increase in their consumption, there was less accumulation of visceral and subcutaneous fat [49]. Consumption of a balanced diet (rich in vegetables, fruits, whole grains, and unsaturated fats) associated with the practice of physical exercise helps with the prevention and treatment of obesity [36]. In a study involving 152 adult individuals, it was found that, when there was a greater consumption of vegetables, fruits, and unsaturated fats, body fat, the occurrence of hypertension, and the contraction of triglycerides decreased [50] Regarding carotenoids, studies have shown that their consumption helps in the prevention and treatment of obesity. In a cross-sectional study with nondiabetic obese men and women, it was observed that individuals who consumed a higher concentration of carotenoids from fruits and vegetables had a higher concentration of adiponectin and, therefore, a greater sensitivity to insulin, in addition to a reduction in body weight [47] (Table 1).

Carotenoids can also regulate other parameters involved in obesity, such as insulin resistance and dyslipidemia [14,49]. Another study noted that the concentration of serum carotenoids (β-carotene, α-carotene, and β-cryptoxanthin) was associated with a lower risk of developing dyslipidemia [51]. The correlation was also observed in a cross-sectional study involving 2148 Chinese adults (aged 50–75 years), where individuals with the lowest concentrations of carotenoids, mainly β-carotene, had increased concentrations of total cholesterol and body weights [52].

The reduction of lipid parameters related to dyslipidemia is another point that indicates the possible preventive role of β-carotene in the development of obesity [55]. One study showed that adults supplemented with frozen vegetables (300 g/day for two weeks) had an increase in the total plasma carotenoid concentration, especially β-carotene, which increased by 56%. In addition, with this increase, a lower concentration of total cholesterol was found in the plasma [56].

The supplementation of β-carotene (6.0 g) from algae (*Dunaliella bardawil*) associated with vitamin A (1500 international units (IU)) was also demonstrated to reduce the concentration of total cholesterol in the plasma [45]. Another study found that supplementation with β-carotene-fortified symbiotic food containing 0.05 g of β-carotene (three times/day for six weeks), while reducing the insulin resistance also resulted in reductions in triglycerides, VLDL-c, and the VLDL total/HDL-c ratio [16].

In a randomized, single-blind, placebo-controlled parallel study evaluating adults affected by MetS who consumed a strict diet of carbohydrates and eggs (3 units/day containing 40 µg of β-carotene) or a substitute (containing 697 µg of β-carotene) for 12 weeks, it was found that, for both supplementation groups, a reduction in the concentrations of triglycerides and increases in HDL-c and β-carotene occurred [53].

MetS encompasses a series of metabolic changes that increase the risk of developing cardiovascular diseases and T2DM. It is defined by the presence of three of five established criteria. Among them are the accumulation of abdominal fat, high levels of LDL-c, and low levels of HDL-c [39,49]. The increase in HDL-c caused by β-carotene is fundamental, as it prevents oxidative stress due to its antioxidant and anti-inflammatory properties and, thus, helps in the prevention of MetS, in addition to reducing hyperglycemia [57].

Carotenoid intake during childhood has also been proven to be a necessary point of monitoring, since diet is a factor that is capable of stimulating or assisting in the treatment of obesity. Due to this, it is necessary to encourage the consumption of fruits and vegetables during childhood [36,54,58,59].

The World Health Organization (WHO) [60] estimated that, in 2019, 38.3 million children under five years of age were overweight, with more than eight million records when compared to 2000. A cross-sectional study that evaluated obese children for five years showed that a high consumption of energy-rich foods increased the risk of developing MS. In addition, 60.2% of these children were found to have a higher risk for developing cardiometabolic diseases due to insulin resistance caused by the diet [59].

A randomized double-blind study in obese children (8–11 years) showed that the quality of the diet improved with a mixed supplementation of carotenoids (containing 2000 IU of β-carotene, in addition to other carotenoids) for six months. A relationship between the reduction of body adiposity and the increase in the concentration of β-carotene was observed [15].

Another study revealed that overweight children (boys aged 6–10 years) receiving vitamin supplements based on fruits and vegetables (twice daily for six months) showed an increase in the concentration of β-carotene available in the serum (334%), in addition to a reduced body adiposity and improved insulin resistance [54].

Hereditary burden has also been shown to be a factor directly related to carotenoid concentrations in children. A study of nondiabetic children and adolescents showed that the concentrations of α- and β-carotene were related to hereditary load, as well as being influenced by diet. These authors observed that, when these carotenoids were present in high concentrations in the body, there was a reduction in weight gain and, thus, a reduction in the risk of developing obesity and its comorbidities, like T2DM [55].

## 4. Type 2 Diabetes Mellitus

According to the WHO [61], diabetes mellitus is defined as a chronic and progressive disease characterized by high levels of glucose in the blood, which can lead to serious health complications and even premature death. The cause for the high glucose level in T2DM patients is the inefficiency of β-pancreatic cells, which, consequently, generate a state of resistance to insulin and or lead to a reduction in its secretion. Besides increasing the glucose levels, such a state brings other complications derived from the metabolic decompensation [16,62].

The factors related to its development include metabolic and genetic conditions, as well as eating habits, a lack of physical activity, tobacco use, the consumption of alcohol, and being overweight [61]. Among these, diet is a primordial factor for its prevention. Thus, a diet of high nutritional quality becomes necessary for individuals with T2DM [14].

Among the components of the diet, β-carotene has been demonstrated to play roles in adipogenesis, lipolysis, insulin resistance, and other factors linked to T2DM [14]. A cross-sectional study with Japanese men and women showed that the concentration of β-carotene in the serum was inversely associated with insulin resistance. Of the individuals, men presented the lowest concentrations of β-carotene, and this finding was attributed to the participants’ smoking and drinking habits, as shown in Table 2, and, thus, higher insulin resistance [35].

A similar result was found in another study, in which male smokers presented carotenoid concentrations half as great as those of nonsmokers and, as a consequence, had higher blood glucose levels [63]. The literature cites that tobacco diminishes the plasmatic concentrations of carotenoids due to the induction of oxidative stress, which depletes these components. It has also been shown that tobacco can be associated with the increased degradation and reduced bioavailability of β-carotene [23,64,65].

Another study pointed out that men are more prone to developing T2DM due to their eating habits (higher intake of calories) and lower levels of physical exercise. In the Korea National Health and Nutrition Examination Survey, it was observed that the proportion of men affected by diabetes was 60.1%, although no correlation of its development with the consumption of carotenoids was shown [66]. Nevertheless, in another study, it was observed that Japanese men and women who consumed higher quantities of plant sources of carotenoids presented higher concentrations of serum β-carotene and lower insulin resistance, indicating a possible protective role of this antioxidant for β-pancreatic cells [68].

In a population-based cross-sectional study evaluating healthy Japanese adults, it was observed that women consumed higher quantities of plant sources of carotenoids and, as a consequence, had higher concentrations of carotenoids in the serum and skin. Of the carotenoids present in the skin, β-carotene was present in the highest concentration (0.517 µg/mL). Those authors found that the levels of carotenoids in the skin were associated with a lower body mass index, blood pressure, index of insulin resistance, and triglyceride concentration, as well as an increase in HDL-c levels [69].

As previously mentioned, insulin resistance is a key point in T2DM. It is characterized as an insufficient response of tissues to the effects of circulating insulin. The state of hyperglycemia leads to the increased production of reactive species of oxygen, thus inducing the inflammatory response, as well as having a direct role in the development of MetS [62,66,70]. This occurs since, in T2DM, macrophages and neutrophils, which liberate active species of oxygen and proinflammatory cytokines such as TNF-α and IL-1β, are activated, causing damage at the cell level [70,71].

With the initiation of oxidative conditions, there is a worsened secretion of insulin and/or increased resistance due to the damage caused in the β-pancreatic cells, aggravating the severity of T2DM [64,70]. Oxidative stress also reduces the capture of glucose by the muscles and adipose tissue [46].

A study showed that individuals under conditions of oxidative stress presented increased fastening concentrations of triglycerides and glycemia, as well as lower concentrations of HDL-c. Qualitatively, these Korean individuals consumed more food sources of carotenoids when compared with healthy individuals; however, they were shown to be recruited to remove the reactive oxygen and nitrogen species [17]. Furthermore, a high content of triglycerides is commonly observed in individuals with T2DM, which promotes the development of hyperglycemia due to the activation of hepatic gluconeogenesis [39].

Hyperglycemia is also responsible for diminishing the uptake of carotenoids since, in this condition, the organism suppresses the production of bile, a component that is fundamental for the absorption of liposoluble vitamins, as is the case for this group of antioxidants [65]. A cross-sectional study demonstrated that individuals affected by diabetes presented the lowest serum concentrations of carotenoids, with the concentration of β-carotene having a significant difference and showing a correlation with a higher risk for developing T2DM [64].

Insulin resistance can also be caused by the exposure to phthalates, which can alter glucose metabolism. A study showed that di-2-ethylhexyl phthalate (DEHP) when present in the urine of adults was positively associated with insulin resistance. Those authors observed that this effect was reduced when the level of β-carotene increased, thus indicating its protective role in this situation [67]. β-carotene can also modulate the metabolisms of lipids and carbohydrates, resulting in better activity of the β-pancreatic cells and improving the conditions of hyperglycemia. With regulation in the functions of the β-pancreatic cells, the stimulus for the secretion of insulin occurs, lipidic metabolism is regulated, and the condition of oxidative and inflammatory stress is alleviated [16,51,62].

Supplementation with β-carotene has also shown to be beneficial for the treatment of T2DM [64]. In a double-blinded, placebo-controlled, crossover clinical trial, adult individuals (35–70 years) in Iran, when supplemented with β-carotene, fortified a symbiotic food containing 0.05 g of β-carotene (three times a day for six weeks), presented an improved insulin metabolism, and reduced insulin resistance. Such effects were attributed to a possible impact on genic expression that resulted in β-carotene inhibiting the production of the free radicals and preserving the receptors of insulin [16].

The daily consumption of carotenoids has also been shown to be effective in reducing the incidence of T2DM over long periods of time. In the European Prospective Investigation into Cancer and Nutrition—Netherlands Study, the evaluation of 37,846 adults for 10 years indicated that a mean intake of 10 g/day of carotenoids, mainly involving a high intake of β-carotene, is protective against T2DM [49]. Besides β-carotene, other carotenoids, such as α-carotene and β-cryptoxanthin, are associated with a reduction in hyperglycemia [65].

Thus, it is possible that β-carotene exerts a protective role against the development of T2DM and can also be used for its treatment. Such a role is attributed to its antioxidant and anti-inflammatory activity. Since, in T2DM, an increase in reactive oxygen species is common due to hyperglycemia and a reduction in antioxidant defenses, this compound would act to regulate the redox balance, avoiding the formation of new reactive oxygen species and inhibiting lipid peroxidation in cell membranes [57]. In addition, β-carotene has been demonstrated to act in other diseases such as obesity, a condition that is directly associated with T2DM [35,49,60,62,70].

## 5. Conclusions

Considering the points made, we conclude that β-carotene has a vital influence in the prevention of diseases such as obesity and T2DM. Such positive effects are associated with its antioxidant capacity, since this component has the ability to prevent and control the oxidative stress that is found in these diseases.

In view of all the results, further studies are still needed in order to understand more deeply what happens during these processes. Finally, we suggest that the consumption of this antioxidant, as well as others, should be investigated, and unconventional foods that meet the daily recommended needs should be identified, as a way of preventing chronic nontransmissible diseases.

## Figures and Tables

**Table 1:** Effects of carotenoids on obesity.

Host	Study	Effects on Obesity
Obese nondiabetic patients (*n* = 108) [47]	24-h recall and Bilnut software	↑ intake of carotenoids = ↓ body weight, and insulin resistance; ↑ [ ] of carotenoids = ↑ adiponectin plasma
Humans (*n* = 374 men) [49]	FFQ	↑ intake total carotenoids, β- and α-carotene, and lycopene = ↓ visceral and subcutaneous fat mass
Humans (*n* = 1073) [51]	FFQ	↑ intake α and β-carotene and β-cryptoxanthin = ↓ dyslipidemia
Humans (*n* = 2148) [52]	FFQ	↓ [ ] carotenoids (β-carotene) = ↑ [ ] glucose, TC and body weight
Humans affected by diabetes (*n* = 51) [16]	(1) β-carotene fortified symbiotic (2) control food for 6 weeks	(1): ↓ triglycerides, VLDL-c levels, and total-/HDL-c ratio
Humans affected by MetS (*n* = 37) [53]	(1) low-carb diet + eggs (3 units/day with 40 µg of β-carotene); (2) low-carb diet + egg substitute (697 µg β-carotene)	(1,2): ↓ triglycerides and ↑ HDL-c
Children (*n* = 20) [15]	(1) Mixed carotenoids supplement (2) Placebo 2x/day for 6 months	(1): ↑β-carotene = ↓ visceral and subcutaneous adipose tissue; (1): ↑ β-carotene = ↑ total adiponectin and high-molecular-weight adiponectin
Prepubertal boys (*n* = 30) [54]	(1) FVJC lean; (2) FVJC obese; (3) Placebo lean; (4) Placebo obese for 3x/day for 6 months	(1 and 2): ↑ [ ] β-carotene (2): improved lipidic parameters and ↓ triglycerides; ↓ adiposity and insulin resistance (4): ↑ triglycerides and↓ HDL-c
WT and Bcmo1-/- mice (*n* = 24) [13]	(1) WT control diet; (2) WT β-carotene-enriched diet; (3) Bcmo-/- control diet; (4) Bcmo-/-β-carotene-enriched diet for 14 weeks.	(2): ↓ body adiposity and size of adipocytes; (2,4): ↑ [ ] of β-carotene in serum and white adipose tissue
Male apoE-/- mice (*n* = 39) [45]	(1) VAD; (2) VAD + 1500 IU vitamin A/kg; (3) VAD + 6 g β-carotene (algal powder); (4) VAD + 1500 IU vitamin A/kg + 6 g β-carotene.	(2): ↓ plasma cholesterol (4): ↑ [ ] of plasma β-carotene and↓ plasma cholesterol

Abbreviations: ↑ = increase, ↓ = decrease, [ ] = concentration, WT = wild-type, FVJC = encapsulated supplement of fruit and vegetable juice concentrate, HDL-c = high-density lipoprotein cholesterol, FFQ = food frequency questionnaire, TC = total cholesterol, VAD = vitamin A deficiency, IU = international units, and VLDL-c = very-low-density lipoprotein cholesterol.

**Table 2:** Effects of carotenoids on T2DM parameters in humans.

Host	Study	Effects in T2DM
Humans (*n* = 951) [35]	FFQ	↑ β-carotene intake = ↓ insulin resistance
Humans (*n* = 4390) [66]	FFQ	No significant association was found between carotene intake and T2DM
Humans (*n* = 747) [64]	FFQ (1) Nondiabetes mellitus (2) Diabetes mellitus	↓ [ ] β-carotene = ↑ fasting glycemia (2): ↓ [ ] carotenoids in serum (β-carotene) = ↑ risk for DM
Humans (*n* = 1605) [67]	Nutritional and biochemical analyses	↑ [ ] β-carotene = ↓ insulin resistance induced by DEHP
Humans affected by diabetes (*n* = 51) [16]	(1) β-carotene fortified symbiotic (2) control food for 6 weeks	(1): ↓ insulin resistance ↑ plasma nitric oxide and glutathione
Humans (*n* = 37,486) [49]	FFQ (Mean total carotenoid intake of 10 g/day)	↑ consumption of α and β-carotene = ↓ risk for T2DM
Humans (*n* = 951) [68]	Self-administered questionnaire	↑ [ ] β-carotene in serum = ↓ insulin resistance
Humans (*n* = 811) [69]	Self-administered questionnaire	↑ [ ] carotenoids in skin and serum in women = ↓ body mass index, blood pressure, index of insulin resistance and triglycerides
Humans (*n* = 1229) [17]	(1) Health (2) Oxidative stress conditions via Recommended Food Score	(1): ↑ [ ] plasma carotenoids (β-carotene and others) (2): ↑ consumption of carotenoids but ↓ [ ] in plasma. (2): carotenoids were recruited to fight oxidative stress (2): ↑ [ ] fasting triglycerides and glycemia

Abbreviations: FFQ = food frequency questionnaire, ↑ = increase, ↓ = decrease, [ ] = concentration, T2DM = type 2 diabetes mellitus, and DEHP = di-2-ethylhexyl phthalate.
